# A Vortioxetine–Glycyrrhizic Acid Supramolecular Complex: Synthesis and Cellular Effects on Microglial and Blood Cells Under Inflammatory and Glucocorticoid Challenge

**DOI:** 10.3390/biomedicines14071540

**Published:** 2026-07-09

**Authors:** Julia N. Khantakova, Elizaveta S. Meteleva, Yulia A. Ryabushkina, Nikolay E. Polyakov, Arina O. Degtyareva, Rasha Salman, Alexsander V. Dushkin, Natalya P. Bondar

**Affiliations:** 1 Institute of Cytology and Genetics, Siberian Branch of Russian Academy of Sciences (SB RAS), Prospekt Lavrentyeva 10, 630090 Novosibirsk, Russia; ryabushkina@bionet.nsc.ru (Y.A.R.); degtyareva@bionet.nsc.ru (A.O.D.); salmanrasha030@gmail.com (R.S.); 2 Institute of Solid State Chemistry and Mechanochemistry, Siberian Branch of Russian Academy of Sciences (SB RAS), Kutateladze St., 18, 630090 Novosibirsk, Russia; solidmete@mail.ru (E.S.M.); dushkin@solid.nsc.ru (A.V.D.); 3 Institute of Chemical Kinetics and Combustion, Siberian Branch of Russian Academy of Sciences (SB RAS), Institutskaya St., 3, 630090 Novosibirsk, Russia; polyakov@kinetics.nsc.ru; 4 Faculty of Natural Sciences, Novosibirsk State University, Pirogova St. 2, 630090 Novosibirsk, Russia

**Keywords:** vortioxetine, glycyrrhizic acid, supramolecular complex, glucocorticoid receptor, neuroinflammation, microglia, depression, HPA axis

## Abstract

**Background**: Depression is a severe disorder associated with hypothalamic–pituitary–adrenal (HPA) axis dysregulation and neuroinflammation, and which restrains the efficacy of conventional antidepressants. Vortioxetine is a multimodal antidepressant with potential immunomodulatory properties. Glycyrrhizic acid (GA) is a natural compound derived from licorice root that exhibits anti-inflammatory activity and modulates glucocorticoid signaling. We hypothesized that a supramolecular complex of vortioxetine with GA (Vort:Na_2_GA) would exert synergistic effects on inflammatory and glucocorticoid pathways. **Methods**: Vortioxetine compositions with Na_2_GA were prepared using a mechanochemical method. Cytotoxicity, anti-inflammatory property, and glucocorticoid receptor (GR) signaling pathway modulation of the complex were evaluated in vitro using SIM-A9 microglial cells. Additionally, a 7-day oral administration study in intact female C57BL/6 mice was conducted to evaluate the effects on peripheral blood cells. **Results**: The Vort:Na_2_GA complex improves the solubility of the parent drug while increasing its stability and permeability. Furthermore, the resulting complex exhibits reduced cytotoxicity, particularly under glucocorticoid challenge. In SIM-A9 microglial cells, the Vort:Na_2_GA complex upregulated expression of *Nr3c1* and *Nr1d1* genes without activating canonical GR target genes (*Fkbp5* and *Gilz*) and partially reversed dexamethasone-induced glucocorticoid resistance. In vivo, the complex reduced the percentage of inflammatory Ly6C^high^ monocytes and preserved dexamethasone-induced *Gilz* expression in peripheral blood cells, indicating protection against stress-induced glucocorticoid resistance. **Conclusions**: The Vort:Na_2_GA supramolecular complex enhances the physicochemical and pharmacological profile of vortioxetine, reduces inflammation-associated myeloid cell populations, and preserves glucocorticoid sensitivity. These findings support its further evaluation as a potential therapeutic agent for depressive disorders with inflammatory and HPA axis-related components.

## 1. Introduction

The global prevalence of depression is rising annually, exacerbating the economic and social burden. Despite numerous studies, modern depression treatments are not always effective [1]. This limited therapeutic efficacy is partly due to the heterogeneous pathogenesis of the disorder, which cannot be fully described by any single pathophysiological framework, such as monoaminergic deficiency, dysregulation of the hypothalamic–pituitary–adrenal (HPA) axis or the immune system, or impaired neuroplasticity. Consequently, modern pharmacotherapy for depression increasingly shifts toward combination regimens or the development of multimodal agents capable of targeting multiple components of the disorder’s pathogenesis.

It is known that approximately half of depressive disorder cases exhibit activation of the glucocorticoid system, manifested by elevated levels of cortisol, corticotropin-releasing hormone (CRH), and adrenocorticotropic hormone (ACTH) [2,3]. Furthermore, patients with severe depressive disorder exhibit impaired HPA axis suppression following dexamethasone administration [4]. On the other hand, treatment-naïve patients exhibit elevated levels of pro-inflammatory cytokines and expression of the inflammasome protein (NLRP3) [5]. Many antidepressants can reduce cytokine levels in the blood of depressed patients [6], and may even improve behavioral parameters during acute inflammation [7]. However, following antidepressant treatment, a reduction in pro-inflammatory marker levels was observed only in treatment responders, whereas levels continued to rise in non-responders [5,8,9]. Consequently, there is a profound clinical need for pharmacological agents capable of not only restoring neurotransmitter balance but also normalizing immunological parameters and HPA axis dysregulation.

Vortioxetine is a novel multimodal antidepressant that has recently been widely used in clinical practice [10,11]. Its therapeutic effect is achieved through a combination of serotonin transporter inhibition and modulation of the activity of several 5-HT receptor types [12]. Vortioxetine has demonstrated potential immunomodulatory properties associated with antioxidant activity [13], reduced cytokine response upon inflammation induction in human macrophages [14], and increased levels of the anti-inflammatory cytokine IL-4 in the rat brain [15]. In tryptophan depletion-induced depression in rats, vortioxetine reduced both corticosterone levels and serum IL-6 levels [16], suggesting a favorable profile of this antidepressant-like effect on HPA axis function as well. Clinical studies demonstrated that adjunctive celecoxib (a nonsteroidal anti-inflammatory drug) with vortioxetine showed no short-term benefit over placebo during the initial 6-week treatment phase [17]. However, during extended follow-up (up to 35 weeks), patients with elevated baseline inflammation who received celecoxib exhibited the most pronounced long-term clinical improvement [18]. These results suggest that the additional anti-inflammatory component provided by celecoxib, when added to the antidepressant, may confer a delayed positive effect in patients with elevated baseline inflammation, rather than representing an immediate, universally effective enhancement strategy.

Thus, combined treatment strategies for depression offer significant therapeutic advantages over monotherapy and support the development of multimodal agents that simultaneously target key pathogenetic pathways: the neurotransmitter system, immune inflammation, and HPA axis regulation.

A promising approach to developing such drugs is the use of natural compounds with established biological properties as carriers for antidepressants. These systems not only improve pharmacokinetic parameters, such as solubility and bioavailability, but can also provide a synergistic therapeutic effect due to the inherent pharmacological activity of the carrier [19]. In particular, glycyrrhizic acid (GA), the main triterpene saponin of licorice root (*Glycyrrhiza glabra*), possesses a broad spectrum of pharmacological activity, including anti-inflammatory, antioxidant, immunomodulatory, and hepatoprotective effects [20,21]. Due to its amphiphilic structure, GA can form molecular complexes with hydrophobic compounds, significantly improving their water solubility and bioavailability [19,22]. Importantly, even at high concentrations, GA exhibits a favorable safety profile and demonstrates no significant cytotoxicity [23,24]. In mouse models, GA has been shown to improve cognitive functions in aged animals and increase the numbers of T and B cells in the blood and spleen, without affecting the NK cell pool [25].

Regarding the molecular mechanisms relevant to antidepressant therapy, GA modulates the expression of pro-inflammatory cytokines [26], activates the PI3K/Akt/GSK3β signaling pathway, thereby reducing cytokine production, and inhibits the DNA-binding activity of nuclear factor kappa-B (NF-κB) [24,27]. Beyond its anti-inflammatory effects, GA can bind to glucocorticoid (GR) and mineralocorticoid receptors, although its affinity is significantly lower than that of dexamethasone or aldosterone [28]. GA has been shown to enhance glucocorticoid-induced activation of the GR [29], while not affecting the number of receptors in the cytoplasm but significantly reducing the level of the GR-associated chaperone HSP90 [30]. Therefore, GA can not only improve the pharmacokinetics of antidepressants but also independently modulates GR activity and suppresses inflammation, making it a potentially valuable component for the combination therapy of depressive disorders associated with inflammation and HPA axis dysfunction.

We hypothesize that a supramolecular complex of GA and the multimodal antidepressant vortioxetine will provide a synergistic effect on monoaminergic targets (via vortioxetine), on the inflammatory component of the disorder (via the combined action of both components), and on the HPA axis, while simultaneously increasing the bioavailability of the primary drug.

The aim of this study was to obtain a complex of vortioxetine with glycyrrhizic acid, to characterize its physicochemical properties, and to compare the biological effects of this compound on cell lines and intact animals with those of the pure vortioxetine. To obtain the complex, we used a solid-state mechanochemical approach. This method enables the synthesis of compounds without the use of solvents or liquid phases, making it possible to obtain substances that lack common solubility in any solvent. Moreover, it enhances the chemical stability of the components during the manufacturing process. The approach is based on the principle of incorporating biologically active molecules into intermolecular complexes, exploiting the properties of hydrophilic polymers and plant-derived vesicular systems [31]. For the obtained vortioxetine–glycyrrhizic acid complex, solubility and stability were evaluated, along with a comprehensive study of its biological activity in vitro (using microglial SIM-A9 cell line) and in vivo (in C57BL/6 mice). Special attention was paid to comparing the anti-inflammatory properties, effects on glucocorticoid receptor signaling pathways under conditions modeling glucocorticoid excess, cytotoxicity, and permeability of the complex with those of pure vortioxetine. The obtained results allow us to evaluate the potential of the developed supramolecular complex as a promising agent for the therapy of depressive disorders associated with inflammation and HPA axis dysfunction.

## 2. Materials and Methods

### 2.1. Drugs

Vortioxetine hydrobromide (1-(2-((2,4-Dimethylphenyl)thio)phenyl)piperazine hydrobromide) of pharmaceutical grade (CAS Number 960203-27-4, BLD Pharmatech, Shanghai, China) was used without further purification. Disodium salt of glycyrrhizic acid (disodium glycyrrhizinate, Na_2_GA) was purchased from Shaanxi Sciphar Biotechnology Co., Ltd., Xi’an, China. All other chemicals were of analytical grade and used without further purification.

### 2.2. Mechanochemical Preparation of Vortioxetine Compositions with Na_2_GA

Vortioxetine and Na_2_GA at weight ratios of 1:5, 1:10, 1:20, and 1:40 were loaded into the drum of a roll mill VM-1 with an internal volume of 300 mL and filled with 600 g of 22 mm diameter steel balls. The drum rotation speed was 157 rpm. The total processing time was 16 h. Samples were collected at 2, 4, 6, 8, and 16 h and analyzed for vortioxetine solubility.

### 2.3. Analysis of Vortioxetine by HPLC

The initial vortioxetine and the obtained complexes were analyzed for the content of the active substance (vortioxetine) and its solubility in water from the obtained compositions on a chromatograph Agilent 1200 with a Zorbax Eclipse XDB-C18 column (Agilent Technologies, Santa Clara, CA, USA), 4.6 × 150 mm; column temperature +30 °C; diode array detector. An acetonitrile and acetate buffer system with a pH of 3.4 (40:60) was used as an eluent with a flow rate of 1 mL/min. The detection wavelength was 225 nm. The concentrations of vortioxetine were determined relative to its specially prepared solutions in ethanol. Identification of vortioxetine peaks in chromatograms was carried out on the basis of retention times, as well as by the spectral ratio method, compared with the initial vortioxetine.

### 2.4. Gel Permeation Liquid Chromatography

The molecular weight distribution (MWD) of the samples was studied using gel permeation chromatography (GPC). MWD was determined on an Agilent 1200 chromatograph (Agilent Technologies, Santa Clara, CA, USA) with a PL aquel-OH 40, 300 × 7.5 mm column. The column temperature was +30 °C. The detector was refractometric. A 0.02% aqueous solution of NaN3 was used as a solvent and eluent; the test solution was prepared with a concentration of 0.2% by substance. The flow rate was 1 mL/min. Calibration was performed using Sigma-Aldrich dextran standards with molecular weights of 12, 50, 80, 150, 270, and 410 kDa. Agilent GPC Date Analysis software (version A.02.01) was used to process the obtained results and calculate MWD.

### 2.5. Determination of Water Solubility of Vortioxetine from Its Complexes

An “excess” sample of the composition powder (calculated at 5 g/L for vortioxetine) was placed in a 25 mL flat-bottomed flask, 2.5 mL of distilled water was added, and the mixture was mixed in an orbital shaker (200 rpm) for 2 h at +37 °C. The resulting suspension was filtered and analyzed on a chromatograph.

### 2.6. Stability Test

To study the stability of vortioxetine in mechanochemically prepared compositions, the original vortioxetine and its mechanically processed products were stored separately in hermetically sealed containers at 40 °C for 30, 60, and 90 days. To determine the vortioxetine content in the vortioxetine/Na_2_GA compositions, all powder samples were completely dissolved in 25 mL of ethanol and analyzed by HPLC.

### 2.7. NMR Spectroscopy

The ^1^H NMR relaxation method was used to confirm the formation of intermolecular complexes. ^1^H NMR spectra were recorded on a Bruker AVANCE III 500 spectrometer (Bruker BioSpin, Rheinstetten, Germany) at 500 MHz and 30 °C. T2 phase relaxation times were measured using the standard Kara-Purcell-Mehboom-Gill (CPMG) sequence: P1(90°)–(τ–P2(180°)–τ)n—registration, where τ = 0.5 ms is a fixed time delay, and n varied from 0 to 2000. Stock solutions of the complexes were prepared by dissolving the solid dispersion in D2O at a concentration of 1% (10 mg/mL), stirred on a magnetic stirrer for 1 h, and centrifuged to remove undissolved precipitate.

### 2.8. Powder X-Ray Diffraction (XRD)

X-ray diffraction analysis of solid complexes was carried out on a D8 Advance Bruker powder diffractometer (Bruker AXS, Karlsruhe, Germany).

### 2.9. Scanning Electron Microscopy (SEM)

The photographs were taken using a Hitachi TM-1000 microscope (Hitachi High-Technologies Corporation, Tokyo, Japan). Gold sputtering was applied to the samples using a JEOL JFC-1600 auto fine coater (JEOL Ltd., Tokyo, Japan). The coating thickness was 15 nm.

### 2.10. Cell Culture

The SIM-A9 microglial cell line (ATCC CRL-3265) was cultured in T75 flasks (Jet Biofil, Guangzhou, China) in DMEM/F12 medium containing 10% fetal bovine serum (FBS), 5% horse serum (HS), and 1% penicillin-streptomycin (all reagents BioloT, Saint-Petersburg, Russia) [32]. Cells were passed upon reaching 70–80% confluence using a 0.25% Trypsin-Versene solution (BioloT, Saint-Petersburg, Russia) for 5 min at +37 °C. Prior to use in experiments, the cells were passed three times. Only the adherent fraction of SIM-A9 cells was used in all experiments.

### 2.11. Cell Viability Assay

To assess any potential toxicity of the drugs, cell viability of the SIM-A9 cells was evaluated using the CCK-8 assay (Cell Counting Kit-8, Elabscience, Wuhan, China). The cells were harvested using 0.25% Trypsin-Versene solution (BioloT, Saint-Petersburg, Russia) and then seeded in 96-well plates (5 × 104 cells). Cells were incubated with vortioxetine and complexes with Na_2_GA (Vort:Na_2_GA, weight ratio 1:5, 1:10, 1:20) in a concentration range between 75 nM and 15 μM for 24 h incubation at 37 °C with 5% CO2; four hours before the end of incubation, 10 μL CCK-8 assay solution was added to the medium to assess the metabolic activity of cells. Then, the absorbance at 450 nm was measured with a microplate reader VICTOR3 (Perkin Elmer, Shelton, CT, USA). Cell viability was calculated as follows: [(absorbance of samples) − (absorbance of background)]/[(absorbance of control) − (absorbance of background)] × 100%, where background represents the mixture of CCK-8 reagent and DMEM/F12 without cells.

To model HPA axis activation, SIM-A9 cells were first pre-conditioned with 25 nM dexamethasone for 72 h. Subsequently, the cells were incubated for 24 h at 37 °C under 5% CO_2_ with 75 nM vortioxetine, either alone or as part of Vort:Na_2_GA complexes (weight ratios 1:5, 1:10, 1:20). Cellular metabolic activity was then assessed using the CCK-8 assay.

### 2.12. Design of In Vitro Experiments

To compare the effects of vortioxetine and its complexes on target gene expression, we used the SIM-A9 microglial cell line. The 48 h time point was selected as a short-term screening window to compare the activities of different formulations. This duration allowed us to reliably capture early responses in microglial cells without the complexity of long-term adaptive changes.

Cells were seeded in 48-well plates at 10^6^ cells/mL and incubated for 48 h at 37 °C/5% CO_2_ with the test compounds vortioxetine (75 nM) and Vort:Na_2_GA compositions (1:5, 1:10, and 1:20 weight ratios). The vortioxetine concentration was kept constant at 75 nM in all complexes. Stock solutions were prepared as follows: vortioxetine HBr was dissolved in 100% DMSO and then diluted with culture medium to a final DMSO concentration of 0.001%. Vort:Na_2_GA complexes were first dissolved in culture medium, followed by DMSO addition to achieve the same final vehicle concentration (0.001% DMSO). Control wells treated medium containing 0.001% DMSO.

To evaluate the modulatory effect of the compounds on inflammation, lipopolysaccharide (LPS, *E. coli* O55:B5, Sigma-Aldrich, MO, USA) was added to half of the wells at 1 µg/mL, 24 h after the start of drug incubation. Cells were harvested 24 h post-LPS addition (48 h total drug exposure).

In a separate experiment modeling HPA axis activation, SIM-A9 cells were pre-conditioned with 25 nM dexamethasone for 72 h. Subsequently, cells were seeded and treated with the drugs for 48 h using the same protocol, including the LPS challenge in a subset of wells.

After incubation, the medium was removed, and cell lysates were collected in ExtractRNA reagent (Evrogen, Moscow, Russia) for RNA isolation and subsequent gene expression analysis.

### 2.13. Caco-2 Cells Permeability Assay

The Caco-2 cell line is a standard in vitro model for evaluating drug permeability through the intestinal epithelium, providing a reliable prediction of in vivo absorption [33]. Caco-2 cells ([Caco2] ATCC HTB-37) were cultured in DMEM/F12 medium supplemented with L-glutamine (BioloT, Saint-Petersburg, Russia), 20% fetal bovine serum (Technozerg, Moscow, Russia), 100 U/mL penicillin, and 100 μg/mL streptomycin (BioloT, Saint-Petersburg, Russia). Cells were maintained at 37 °C in a 5% CO_2_ atmosphere.

For permeability assays, freshly trypsinized cells were seeded onto permeable filter inserts (0.4 μm pore size, 0.33 cm^2^ surface area, Jet Biofil, Guangzhou, China) placed in 24-well culture plates at a density of 2.6 × 10^5^ cells/cm^2^. The culture medium was replaced every two days. After 21 days, which is required for the cells to differentiate into a monolayer resembling small intestinal enterocytes, transepithelial electrical resistance (TEER) was measured using a Millicell ERS-2 (Merck Millipore, Darmstadt, Germany) epithelial voltmeter to assess monolayer integrity. Inserts with TEER values ≥ 400 Ω·cm^2^ were considered acceptable for the experiments.

Vortioxetine and the Vort:Na_2_GA 1:20 complex were dissolved in Hank’s balanced salt solution (BioloT, Saint-Petersburg, Russia) at a concentration of 100 ng/μL based on vortioxetine content. The final solutions contained 0.1% DMSO. For the transport study, 150 μL of each test solution was added to the apical chamber containing the cell monolayer, while the basolateral compartment was filled with 400 μL of fresh Hank’s solution. At least five replicates were used per group. Cells were incubated in a thermoshaker at 37 °C with orbital shaking at 80 rpm.

Two experimental series were performed to assess permeability: the first with time points at 15, 30, 45, and 60 min, and the second with time points at 30 min, 1, 2, 4, and 6 h. At each time point, the entire basolateral volume (400 μL) was collected and replaced with an equal volume of fresh Hank’s solution. The concentration of vortioxetine in the collected samples was determined by high-performance liquid chromatography (HPLC) as described previously.

### 2.14. Animals

Adult female mice of the C57BL/6 strain were provided by the Center for Genetic Resources of Laboratory Animals at the Institute of Cytology and Genetics, SB RAS, Novosibirsk, Russia. The animals were housed under standard conditions (12:12 h light/dark cycle, lights on at 7.00 A.M.; feed (pellets) and water were available ad libitum). The mice were weaned at 1 month of age and housed in groups of 8–10 in plastic cages (36 × 23 × 12 cm). Experiments were performed on mice 12 weeks of age. The animal study protocol was approved by the Ethics Committee of the Institute of Cytology and Genetics, SB RAS (protocol #240, 18 July 2025) in conformity with European Communities Council Directive 210/63/EU of 22 September 2010.

### 2.15. Design of In Vivo Experiments

To compare the effects of vortioxetine and its complexes in vivo, intact female mice were treated with the drugs for 7 days. The mice were orally administered vortioxetine (20 mg/kg), Vort:Na_2_GA 1:20 (at the same vortioxetine-equivalent dose), or vehicle daily. The drugs were dissolved in a mixture of water and saline (2:1).

Blood samples were collected from all animals via the retro-orbital sinus to determine blood cell subpopulations and to assess the expression of LPS- and GR-sensitivity target genes in blood cells. During the experiment, blood was collected two times in the morning (10:00 local time): before the start of drug administration and after 7 days of treatment. For each animal, 20 µL of blood was used for cell phenotyping (see Section 2.16). The remaining blood from each animal was then treated ex vivo with LPS (10 pg/µL), dexamethasone (5 ng/µL), or left untreated (control). The incubation was carried out for 2 h at 37 °C with 5% CO_2_. After incubation, red blood cells were lysed using ACK Lysis Buffer (Elabscience, Wuhan, China), and RNA was isolated from the cells.

### 2.16. Flow Cytometry Analysis

The subpopulations of lymphocytes, monocytes, and neutrophils in the blood cell suspension were evaluated using flow cytometry [34]. The following anti-mouse antibodies were used for staining: TCRb-FITC, Ly6C-FITC, CD161/NK1.1-PE, Ly6G-PE, CD11b-PerCP, CD45-PE/Cy7, CD4-PE/Cy7, CD8-APC, and CD19-APC (all from Elabscience, China). The appropriate antibody cocktail was added to 20 µL of the blood cell suspension and incubated for 35 min at room temperature in the dark. Subsequently, 2 mL of 1x ACK Lysis Buffer (Elabscience, China) was added for red blood cell lysis according to the manufacturer’s protocol. The tubes were then centrifuged for 10 min at 4 °C and 300× *g*. The supernatant was discarded, and the cell pellet was resuspended in 200 µL of PBS. Analysis was performed immediately after staining using a BD FACS Aria III-B flow cytometer (BD Biosciences, San Jose, CA, USA). The gate strategy for cell populations is presented in Figures S1 and S2.

### 2.17. RNA Extraction and Real-Time PCR

RNA was extracted from samples in vitro (cells SIM-A9) and in vivo (blood) experiments using the ExtractRNA reagent (Evrogen, Moscow, Russia) following the manufacturer’s instructions. For whole blood samples, pre-lysis of erythrocytes was performed using ACK Lysis Buffer (Elabscience, Wuhan, China) according to the manufacturer’s protocol. RNA quality and quantity were assessed with a NanoDrop 2000 spectrophotometer (Thermo Fisher Scientific, Waltham, MA, USA).

For reverse transcription, 1 µg of RNA was mixed with 100 pmol random hexamer primers in a total volume of 12 µL. The mixture was heated at 65 °C for 5 min and snap-cooled on ice for at least 1 min. The reverse transcription reaction mixture was added to a final concentration of 50 mM Tris-HCl (pH 8.3), 50 mM KCl, 5 mM MgCl2, 10 mM DTT, 1 mM dNTP mix, and 100U of M-MuLV reverse transcriptase (Vector-Best, Novosibirsk, Russia) in a final volume of 20 µL, and the mixture was incubated at 25 °C for 10 min followed by 60 min at 37 °C. Reactions were terminated by heating at 70 °C for 10 min. The reaction was diluted 5-fold to a concentration of 10 ng/µL cDNA.

Real-time PCR was conducted in 20 µL volumes containing 50 mM Tris-HCl (pH 8.5), 50 mM KCl, 0.2 mM dNTPs, 5 mM MgCl2, 0,0125% Tween 20, 5 pmol of forward and reverse primers, 5 pmol of TaqMan probe, 20 ng of cDNA, and 1U of Taq DNA polymerase with antibody-mediated enzyme inhibition (Vector-Best, Novosibirsk, Russia). The protocol included initial heating at 95 °C for 5 min, followed by 39 cycles (denaturation at 95 °C for 15 s, annealing and extension at 60 °C for 20 s). Primers and probes are provided in Table S1. All reactions were performed in duplicate. Gene expression was calculated using the 2^−ddCt^ method.

Reference genes *Eef2* (Eukaryotic translation elongation factor 2), *Pic3c3* (Phosphatidylinositol 3-kinase catalytic subunit type 3), and *Cct5* (Chaperonin Containing TCP1 Subunit 5) were verified using UCSC BLAT to ensure no pseudogenes or non-unique sequences were present. Stability testing of expression in the sample groups was performed with Bio-Rad’s CFX Manager software, Version 3.1, and *Eef2* and *Pic3c3* were chosen for SIM-A9 cells with a group coefficient of variation (CV) of 0.1102 and M-value of 0.3068, and *Eef2* and *Cct5* were chosen for blood cells with a group coefficient of variation (CV) of 0.1147 and M-value of 0.3293.

### 2.18. Statistical Analysis

Each parameter was tested for normality using the Shapiro–Wilk test. Since most data followed a normal distribution, one-way or two-way ANOVA was used, followed by Tukey’s HSD post hoc test for pairwise group comparisons. Statistical significance was set at *p* < 0.05. All analyses were conducted using STATISTICA 6.0 software, and graphical representations were generated using GraphPad Prism version 8.0.1.

## 3. Results

### 3.1. Physicochemical Studies

#### 3.1.1. Solubility

The solubility of vortioxetine and its complex with Na_2_GA was assessed at 2, 4, 6, 8, and 16 h after mechanochemical processing. In all cases studied, an increase in vortioxetine solubility was observed, indicating the high efficacy of Na_2_GA as a complexing agent. The best results were obtained after 2 h of mechanochemical treatment (Table 1). It should be noted that, due to the presence of glycyrrhizic acid in the complex, which acts as a surfactant and can form micelles, the solubility of vortioxetine from the complex could not be determined under classical saturated solution conditions. Increasing the sample weight changes the properties of the solution (e.g., micelle formation), thus preventing the establishment of a true equilibrium saturation. In our experiments, at the highest tested sample weight, the concentration of vortioxetine in the solution reached 10 g/L; however, the actual solubility exceeds 10 g/L, as indicated in the table.

#### 3.1.2. Accelerated Storage

An accelerated storage test of pure vortioxetine and its complexes was conducted over 30, 60, and 90 days (Table 2). It was found that the content of pure vortioxetine decreased slightly, but not significantly (*p* > 0.05), with increasing storage time. In the mechanochemically prepared complexes, the vortioxetine content did not differ significantly from pure vortioxetine at any time point. These results indicate that Na_2_GA increases the stability of the complexes and prevents the oxidation of vortioxetine.

#### 3.1.3. Physico-Chemical Changes in Solid Phases

**Figure 1:**
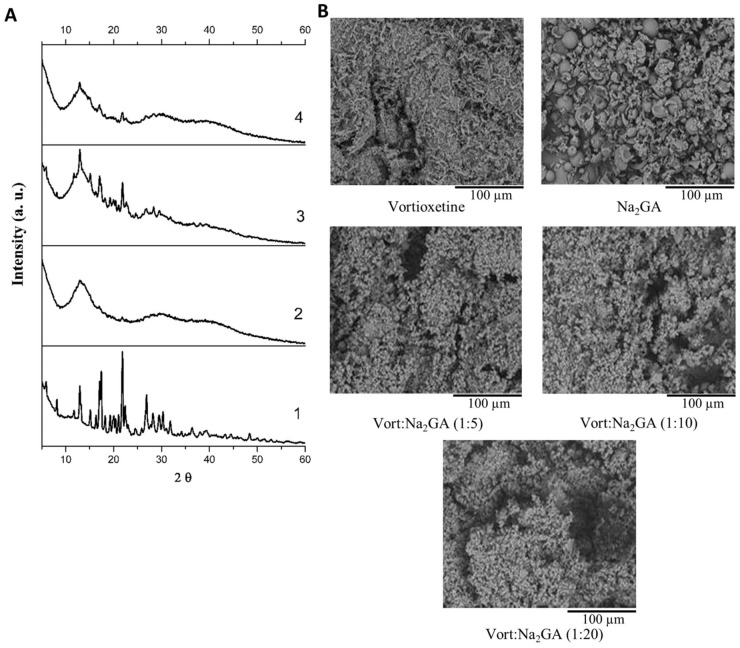
X-ray diffraction and scanning electron microscopy analysis of vortioxetine, Na_2_GA, and their complexes. (**A**) X-ray diffraction patterns of pristine vortioxetine (1), Na_2_GA (2), and the vortioxetine/Na_2_GA 1:20 mixture before (3) and after (4) mechanochemical processing. (**B**) Scanning electron microscopy images of vortioxetine, Na_2_GA, and the dispersed mixtures of vortioxetine/Na_2_GA at ratios of 1:5, 1:10, and 1:20.

X-ray diffraction analysis was performed to evaluate the solid-state phase of the starting materials and the mechanochemically obtained complex (Figure 1A). The diffraction pattern of pristine vortioxetine exhibited sharp reflections characteristic of a crystalline phase. Following mechanochemical treatment with Na_2_GA, the intensity of these reflections markedly decreased, and the peaks broadened, indicating partial amorphization and disordering of the vortioxetine crystal structure.

Scanning electron microscopy was used to assess changes in particle morphology following mechanochemical processing (Figure 1B). Pristine vortioxetine appeared as needle-shaped crystals with an approximate size of 100 µm, consistent with a crystalline material. The starting Na_2_GA consisted of spherical particles ranging from 5 to 50 µm in diameter. After co-processing of vortioxetine and Na_2_GA in a ball mill, the original particles were comminuted, resulting in a polydisperse powder composed of individual particles (10–50 µm) and their aggregates. The aggregates exhibited an amorphous solid-phase morphology, suggesting that vortioxetine molecules are molecularly dispersed within the excess solid carrier.

#### 3.1.4. H^1^ NMR Relaxation Study

It is known that the spin-spin relaxation times of magnetic nuclei in solution are highly sensitive to intermolecular interactions and the diffusion mobility of molecules. This sensitivity arises from changes in the rotational reorientation time (τc) of molecules when they form self-associates or complexes with other molecules. τc can be estimated using the Stokes-Einstein-Debye equation: τc = 4πa^3^η/3kT. When a molecule becomes incorporated into a complex, its proton relaxation times decrease significantly due to reduced diffusion and rotational mobility. Therefore, we used ^1^H NMR relaxation measurements to confirm the formation of intermolecular complexes.

**Figure 2:**
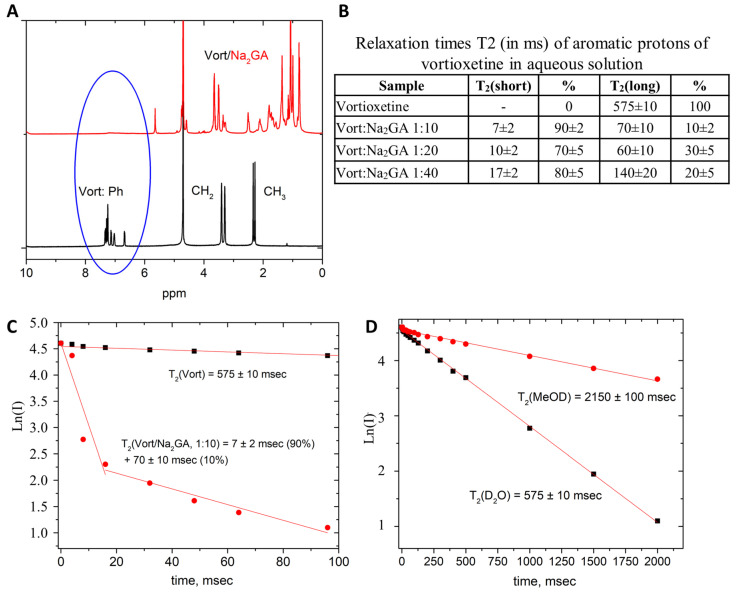
^1^H NMR relaxation study of vortioxetine and Vort:Na_2_GA complexes. (**A**) ^1^H NMR spectra of a D_2_O solution of vortioxetine and a 1% solution of the Vort:Na_2_GA composition obtained by joint mechanical activation of a 1:20 mixture. T = 30 °C. (**B**) Relaxation times T_2_ (in ms) of the aromatic protons of vortioxetine in aqueous solution at 30 °C. Concentration of the Vort:Na_2_GA complexes = 10 mg/mL. For the (1:5) composition, the relaxation time could not be measured due to low solubility. The experiments were performed at least in triplicate, and the results are presented as mean ± SD. (**C**) Time dependence of the echo signal intensity in the CPMG experiment (logarithmic scale) for the aromatic protons of vortioxetine in aqueous solution at 30 °C. Dots represent experimental data; solid lines represent the calculated fit. Concentrations: Vort:Na_2_GA (1:10) = 10 mg/mL, vortioxetine alone = 1 mg/mL. (**D**) Time dependence of the echo signal intensity in the CPMG experiment (logarithmic scale) for the aromatic protons of vortioxetine in aqueous and methanol solutions at 30 °C. Dots represent experimental data; solid lines represent the calculated fit. Concentration of vortioxetine = 1 mg/mL.

In the case of vortioxetine, the most striking observation was the pronounced broadening of its NMR signals in the presence of Na_2_GA (Figure 2A). This broadening indicates not only the incorporation of vortioxetine molecules into micelles but also a substantial increase in micelle size. In this study, we measured the relaxation times of the aromatic protons of vortioxetine, since the signals from aliphatic protons overlapped with those of Na_2_GA. To confirm the formation of associates between vortioxetine and glycyrrhizic acid micelles, we compared the T_2_ relaxation times of protons in pristine vortioxetine and in the mechanochemically obtained complexes prepared by co-milling vortioxetine with Na_2_GA powder at mass ratios of 1:5, 1:10, 1:20, and 1:40 (Figure 2B). A dramatic, approximately 50-fold decrease in the relaxation time of vortioxetine protons was observed for the complex solutions compared to the aqueous solution of the free drug.

Figure 2C illustrates the NMR signal decay kinetics from the CPMG experiment (logarithmic scale) for the aromatic protons of vortioxetine in an aqueous solution of the Vort:Na_2_GA (1:10) complex. The observed decay kinetics for the complex were biexponential. The major contribution (~90%) came from larger aggregates characterized by a short relaxation time of approximately 10 ms, while a minor contribution (~10%) originated from smaller associates with a relaxation time of about 70 ms. With increasing Vort:Na_2_GA ratio to 1:20 and 1:40, the proportion of large aggregates decreased slightly, and their size diminished, as evidenced by the increase in T_2_ relaxation times (Figure 2B). Based on these experiments, we conclude that vortioxetine molecules in aqueous solution are completely incorporated into Na_2_GA micelles or aggregates.

Notably, the T_2_ relaxation time of the aromatic protons of vortioxetine in aqueous solution in the absence of Na_2_GA was 575 ms, which is shorter than typical values for freely tumbling single molecules (1–3 s). We hypothesized that, owing to its hydrophobicity, vortioxetine forms self-associates in aqueous solution. To test this hypothesis, we compared the proton relaxation times of vortioxetine in aqueous and methanolic solutions (Figure 2D). Indeed, in the alcoholic solution, the relaxation time increased substantially from 575 ms to 2470 ms, confirming our hypothesis that vortioxetine forms self-associates in water.

**Figure 3:**
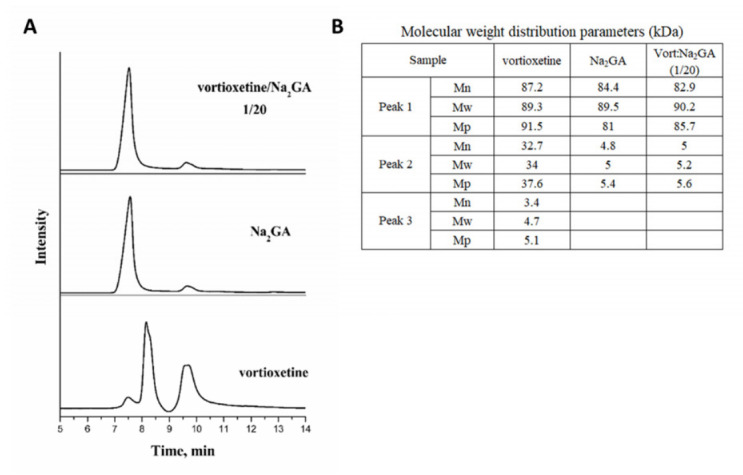
Molecular weight distribution analysis of vortioxetine and its complexes with Na_2_GA by gel chromatography. (**A**) Gel chromatograms of samples in NaN_3_ (0.6% *w*/*w*). (**B**) Molecular weight distribution parameters (kDa): Mn—number-average molecular weight; Mw—weight-average molecular weight; Mp—molecular mass at the peak maximum.

The molecular weight distribution in solution was also examined by gel chromatography (Figure 3A). The results demonstrate that vortioxetine alone forms associates in aqueous solution (Figure 3A,B). However, in the case of the Vort:Na_2_GA complex, larger mixed associates are formed.

### 3.2. Effects of Vortioxetine and Its Complexes on Cell Viability

**Figure 4:**
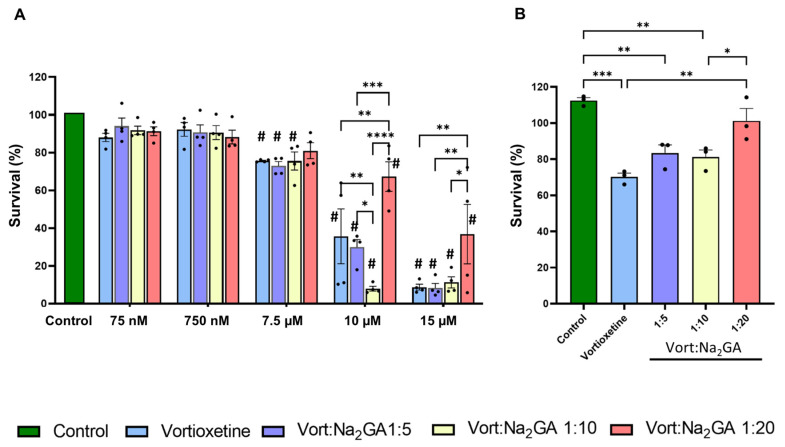
Analysis of the cytotoxicity of vortioxetine and its complexes with glycyrrhizic acid in SIM-A9 cells. (**A**) Cytotoxicity analysis at different concentrations of vortioxetine in the complexes. The x-axis shows the vortioxetine concentration (75 nm–15 μm). (**B**) Cytotoxicity analysis of vortioxetine (75 nm vortioxetine) and its complexes (75 nm based on vortioxetine content) following pretreatment of SIM-A9 cells with dexamethasone (25 nm) for 72 h. Vort:Na_2_GA (1:5), (1:10), and (1:20); complexes of vortioxetine with glycyrrhizic acid at different mass ratios of glycyrrhizic acid. * *p* < 0.05, ** *p* < 0.01, *** *p* < 0.001, **** *p* < 0.0001 for comparisons within the same drug dose; # *p* < 0.05 compared to control. One-way ANOVA with Tukey’s multiple comparisons test. All values were normalized to the control group (cells without drug treatment) without dexamethasone pretreatment. Data are presented as mean ± standard error of the mean (SEM).

None of the tested compounds exhibited cytotoxicity at vortioxetine concentrations of 75 and 750 nM compared to the control (Figure 4A). However, at 7.5 µM, a reduction in cell viability was observed for vortioxetine and the Vort:Na_2_GA (1:5) and (1:10) complexes, but not for the Vort:Na_2_GA (1:20) complex. Exposure to supraphysiological concentrations (10 and 15 µM) induced a dose-dependent decrease in viability across all groups. Notably, this cytotoxic effect was markedly attenuated for the Vort:Na_2_GA (1:20) complex (40% vs. 10% viability reduction). Therefore, the Vort:Na_2_GA (1:20) complex lacks cytotoxic effects on SIM-A9 microglial cells at therapeutically relevant concentrations (up to 7.5 µM). Even at high concentrations, it demonstrates significantly lower cytotoxicity than pure vortioxetine, suggesting that glycyrrhizic acid modulates its toxicological profile.

Pre-treatment with dexamethasone did not affect cell viability compared to the intact control (Figure 4B). Subsequent incubation with vortioxetine or the Vort:Na_2_GA (1:5) and (1:10) complexes, however, reduced viability relative to the dexamethasone-only group. In contrast, the Vort:Na_2_GA (1:20) complex demonstrated a protective effect, maintaining viability at a level comparable to the intact control. Thus, under conditions of elevated glucocorticoids, only the Vort:Na_2_GA (1:20) complex was non-cytotoxic and preserved microglial cell viability. This suggests that glycyrrhizic acid within the complex can mitigate the adverse cellular effects associated with glucocorticoid system hyperactivation.

### 3.3. The Effect of Vortioxetine and the Vort:Na_2_GA Complexes on Pro-Inflammatory Cytokine Gene Expression in Microglial SIM-A9 Cells

**Figure 5:**
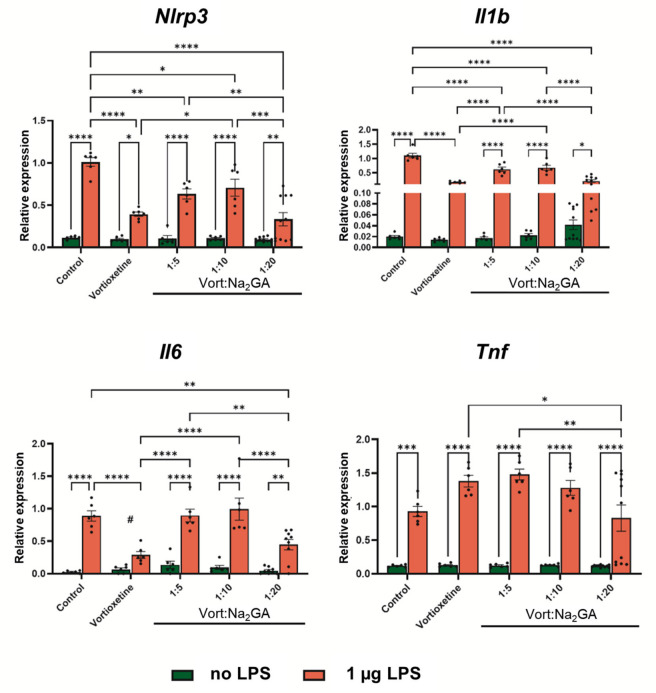
Effect of drug pretreatment (48 h) on the expression levels of pro-inflammatory cytokine genes in microglial SIM-A9 cells following LPS treatment (1 µg, 24 h). Vort:Na_2_GA (1:5), (1:10), (1:20)—complexes of vortioxetine with glycyrrhizic acid at different mass ratios of glycyrrhizic acid. * *p* < 0.05, ** *p* < 0.01, *** *p* < 0.001, **** *p* < 0.0001; two-way ANOVA with Tukey’s multiple comparisons test. Data are presented as mean ± SEM. The dots represent biological replicates.

The anti-inflammatory properties of the compounds were assessed by changes in gene expression in response to LPS stimulation in SIM-A9 cells (Figure 5). Vortioxetine and Vort:Na_2_GA complexes had no effect on the basal expression of the *Il1b*, *Tnf*, and *Il6* genes, which encode pro-inflammatory cytokines, or the *Nlrp3* gene, which encodes the core protein of the inflammasome. A 24 h LPS treatment of cells induced a pronounced immune response and activation of these genes’ expression in all groups. However, pre-treatment of cells with either pure vortioxetine or Vort:Na_2_GA complexes attenuated this response for the *Nlrp3*, *Il1b*, and *Il6* genes compared to the response in SIM-A9 cells without pre-treatment. The Vort:Na_2_GA (1:20) complex exhibited an anti-inflammatory effect similar to that of pure vortioxetine, while the effects of the Vort:Na_2_GA (1:5) and Vort:Na_2_GA (1:10) complexes were less pronounced—attenuation of the LPS induction was not detected for *Il6* gene expression. Pre-treatment with the compounds did not affect the LPS-induced increase in *Tnf* gene expression.

Thus, vortioxetine and its complexes with Na_2_GA demonstrated a selective ability to attenuate the LPS-induced upregulation of key pro-inflammatory mediators (*Nlrp3*, *Il1b*, *Il6*) in microglial cells, with the effect being most pronounced for the Vort:Na_2_GA (1:20) complex and pure vortioxetine.

### 3.4. Effect of Vortioxetine and Vort:Na_2_GA Complexes on Glucocorticoid Receptor Signaling in Microglial SIM-A9 Cells Under Basal and Dexamethasone-Primed Conditions

#### 3.4.1. Effects Under Basal Conditions

**Figure 6:**
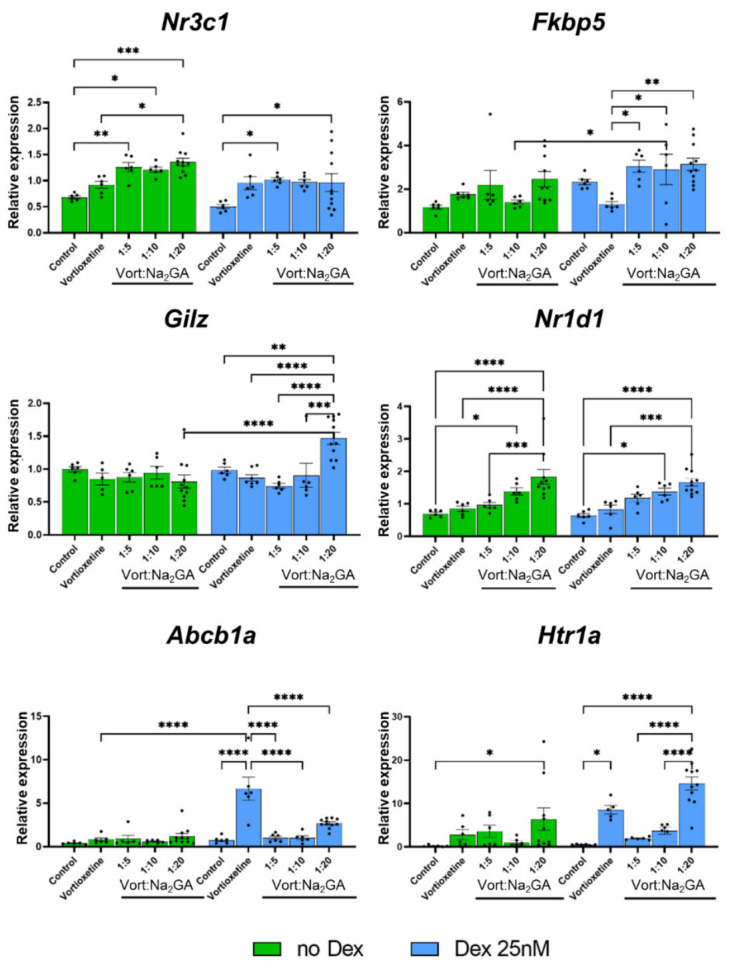
Impact of vortioxetine and its complexes on glucocorticoid pathway gene expression in control (green columns) and dexamethasone-pretreated (blue columns) SIM-A9 cells. Vort:Na_2_GA (1:5), (1:10), (1:20)—complexes of vortioxetine with glycyrrhizic acid at different mass ratios of glycyrrhizic acid. * *p* < 0.05, ** *p* < 0.01, *** *p* < 0.001, **** *p* < 0.0001; two-way ANOVA with Tukey’s multiple comparisons test. Data are presented as mean ± SEM. The dots represent biological replicates.

To evaluate the molecular pathways underlying the effects of vortioxetine and its complexes, murine SIM-A9 microglial cells were treated with the compounds for 48 h. Two-way ANOVA revealed a significant main effect of drug treatment on the expression of all genes studied (Figure 6).

Incubation with Vort:Na_2_GA complexes, but not with vortioxetine alone, significantly increased *Nr3c1* (glucocorticoid receptor, GR) mRNA levels compared to the untreated control. Notably, the Vort:Na_2_GA (1:20) complex induced a more pronounced upregulation of *Nr3c1* than pure vortioxetine. However, the expression of canonical GR target genes (*Fkbp5*, *Gilz* (*Tsc22d3*), and *Abcb1a*) was not altered by any of the tested compounds. Together, these data suggest that the complexes increase *GR* (*Nr3c1*) gene expression without acting as direct GR agonists at the level of canonical target-gene induction under basal conditions.

The expression of *Nr1d1*, a transcription factor associated with anti-inflammatory activity, was significantly increased by the Vort:Na_2_GA (1:10) and (1:20) complexes compared to the untreated control, with the magnitude of upregulation increasing proportionally to the glycyrrhizic acid content. This suggests that these complexes may possess enhanced anti-inflammatory properties.

The expression of the serotonin 1A receptor gene (*Htr1a*), a key pharmacological target of vortioxetine, showed only a modest increase following treatment with the Vort:Na_2_GA (1:20) complex (*p* < 0.05). The lack of a pronounced effect may be explained by the fact that upregulation of *Htr1a* expression and receptor density in the brain are long-term adaptive changes that develop over weeks of chronic in vivo administration, a process that may not be adequately reproduced under short-term (48 h) in vitro conditions.

#### 3.4.2. Effects of Dexamethasone Pretreatment

We next assessed whether the effects of the compounds were dependent on pretreatment with dexamethasone, a glucocorticoid receptor agonist. Prolonged GR activation with dexamethasone models glucocorticoid-induced molecular alterations associated with chronic stress and depression. This approach was used to test the hypothesis that the studied compounds may not only directly modulate GR activity but also correct impairments in GR signaling resulting from excessive or sustained receptor stimulation. Cells were pretreated with dexamethasone for 72 h, after which the test compounds were added for 48 h.

Two-way ANOVA confirmed a significant main effect of dexamethasone pretreatment on the gene expression of *Nr3c1* (*GR*), *Fkbp5*, *Gilz* (*Tsc22d3*), and *Abcb1a*. However, pairwise comparisons revealed that in the control group, dexamethasone induced only a trend toward increased *Fkbp5* gene expression (*p* = 0.07) compared to cells not treated with dexamethasone, indicating reduced GR sensitivity consistent with the development of functional glucocorticoid resistance. In contrast, after dexamethasone pretreatment, the Vort:Na_2_GA (1:10) and (1:20) complexes significantly upregulated *Fkbp5* (*p* < 0.05) and *Gilz* (*p* < 0.001) gene expression, respectively, relative to treatment without dexamethasone, suggesting a partial reversal of glucocorticoid resistance.

Further analysis restricted to dexamethasone-pretreated cells showed that all Vort:Na_2_GA complexes, regardless of their GA ratio, increased *Fkbp5* expression compared to vortioxetine alone, but not compared to the untreated control. For the Vort:Na_2_GA (1:20) complex, an increase in *Gilz* gene expression was observed both compared to the control (*p* < 0.01) and compared to vortioxetine and the other complexes (*p* < 0.001). This indicates a selective enhancement of GR signaling exclusively in the context of an already activated receptor, further supporting the partial reversal of glucocorticoid resistance.

Under dexamethasone pretreatment, Vort:Na_2_GA complexes retained their ability to upregulate *Nr3c1* expression compared to the dexamethasone-only control (*p* < 0.05 for 1:5 and 1:20; *p* = 0.06 for 1:10). The effect on *Nr1d1* expression was also preserved: significant upregulation was observed for the Vort:Na_2_GA (1:10) and (1:20) complexes (*p* < 0.05 and *p* < 0.001, respectively).

Notably, dexamethasone-pretreated cells exposed to vortioxetine alone exhibited a marked increase in *Abcb1a* expression (*p* < 0.001), whereas none of the Vort:Na_2_GA complexes induced such an effect. The *Abcb1a* gene encodes P-glycoprotein, a plasma membrane transporter responsible for xenobiotic efflux and a key mediator of multidrug resistance and blood–brain barrier permeability. Upregulation of *Abcb1a* by vortioxetine may reflect increased P-glycoprotein-mediated efflux and reduced intracellular drug availability. Incorporation of GA into the complexes attenuated vortioxetine-induced *Abcb1a* expression.

Dexamethasone pretreatment also sensitized *Htr1a* expression to vortioxetine: significant upregulation was observed for pure vortioxetine (*p* < 0.05) and, more prominently, for the Vort:Na_2_GA (1:20) complex (*p* < 0.001). These findings indicate that dexamethasone sensitizes microglial cells to the tested compounds, unmasking their ability to modulate Htr1a expression—an effect not observed under basal conditions.

In summary, the developed Vort:Na_2_GA complexes, particularly at a 1:20 ratio, exhibit a favorable profile of gene modulation within glucocorticoid and serotonergic signaling pathways. Under basal conditions, they upregulate *Nr3c1* and *Nr1d1* without activating canonical GR target genes, ruling out direct receptor agonism. Under glucocorticoid challenge, the complexes retain these effects, partially overcome Fkbp5-related resistance, attenuate undesirable *Abcb1a* induction, and enhance *Htr1a* expression—an effect not seen with pure vortioxetine under basal conditions.

Based on its most stable and pronounced effects, favorable safety profile, and ability to modulate key target genes under conditions of glucocorticoid resistance, the Vort:Na_2_GA (1:20) complex was selected for subsequent in vivo studies.

### 3.5. Permeability of Drugs Through a Monolayer of Caco-2 Cells

**Figure 7:**
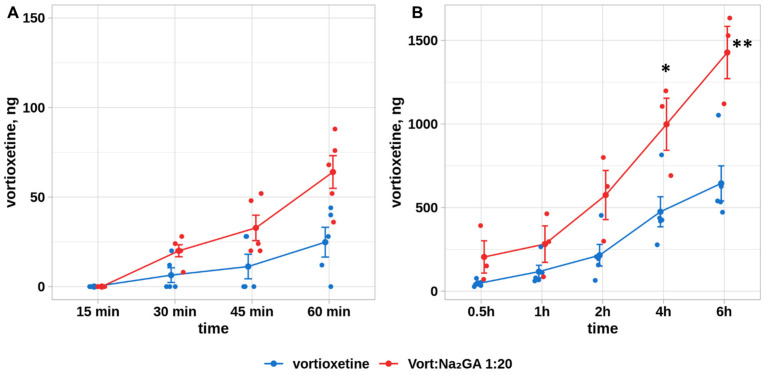
Permeability of drugs through a Caco-2 cell monolayer. (**A**) Amount of vortioxetine diffused across the Caco-2 cell monolayer at 15, 30, 45, and 60 min. (**B**) Amount of vortioxetine diffused across the Caco-2 cell monolayer at 0.5, 1, 2, 4, and 6 h. * *p* < 0.05, ** *p* < 0.01 compared between vortioxetine and the complex; repeated measures ANOVA followed by Tukey’s post hoc test. Data are presented as mean ± SEM. The dots represent biological replicates.

The Caco-2 cell line was routinely cultivated as monolayers on permeable filters for studies of transepithelial drug transport. These cells retain the morphological and functional properties characteristic of intestinal epithelial cells, making them a valuable model to predict drug absorption in vivo. In this experiment, we assessed the transport of vortioxetine and its Vort:Na_2_GA (1:20) complex. The analysis revealed that the diffusion rate of the Vort:Na_2_GA (1:20) complex across the cell monolayer exceeded that of pure vortioxetine (Figure 7), with the difference reaching statistical significance after 4 h of diffusion. These findings suggest that glycyrrhizic acid enhances the apparent intestinal permeability (absorption potential) of vortioxetine, which may translate into faster drug exposure in vivo; this requires confirmation in animal studies.

### 3.6. The Effect of Vortioxetine and Its Complex on Myeloid Blood Cells in Intact Mice

**Figure 8:**
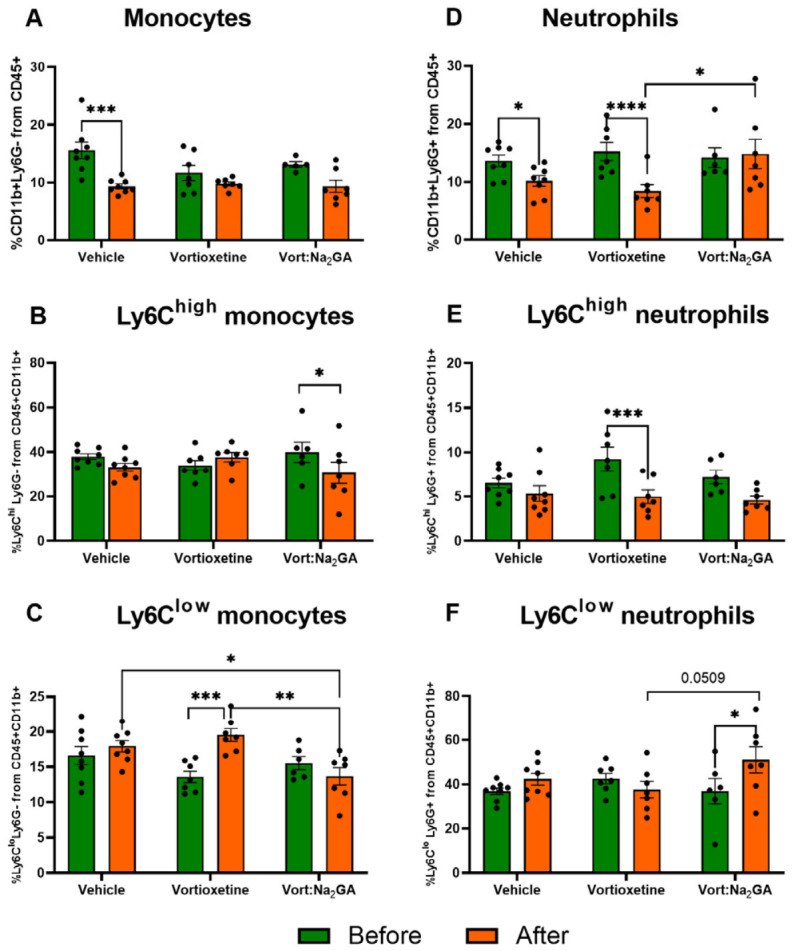
Analysis of myeloid blood cell subpopulations before and after 7 days of drug treatment. (**A**) Percentage of total blood monocytes (CD45^+^CD11b^+^Ly6G^−^). (**B**) Percentage of inflammatory Ly6C^high^ monocytes. (**C**) Percentage of anti-inflammatory Ly6C^low^ monocytes. (**D**) Percentage of total blood neutrophils (CD45^+^CD11b^+^Ly6G^+^). (**E**) Percentage of Ly6C^high^ neutrophils. (**F**) Percentage of Ly6C^low^ neutrophils. Vehicle—group of mice receiving vehicle (N = 8); Vortioxetine—group of mice receiving vortioxetine (20 mg/kg) (N = 7); Vort:Na_2_GA—group of mice receiving the Vort:Na_2_GA (1:20) complex (20 mg/kg based on vortioxetine content) (N = 7). Blood samples were collected from each mouse before the start of treatment and 24 h after the final drug administration. * *p* < 0.05, ** *p* < 0.01, *** *p* < 0.001, **** *p* < 0.0001; two-way repeated ANOVA with Sidak’s multiple comparisons test. Data are presented as mean ± SEM. Dots represent values from individual animals.

Blood cell composition was assessed before the start of treatment and 24 h after the final drug administration. Seven days of oral vehicle administration resulted in a decrease in the proportion of myeloid cells, monocytes (CD45^+^CD11b^+^Ly6G^−^), and neutrophils (CD45^+^CD11b^+^Ly6G^+^), compared to baseline values (Figure 8). In addition, vehicle treatment was accompanied by an increase in CD8^+^ T-killer cells, an elevated CD4^+^/CD8^+^ ratio, and a higher proportion of B-lymphocytes (CD45^+^CD19^+^) (Figure S3). These changes likely reflect cell redistribution associated with stress induced by the feeding procedure and repeated blood sampling [35]. Vehicle administration did not affect the fractions of CD4^+^ T-helper cells or NK cells (Figure S3).

Vortioxetine treatment, similar to vehicle, reduced the total number of neutrophils. However, unlike the control group, vortioxetine also significantly decreased the proportion of immature Ly6C^high^ neutrophils, indicating an additional suppression of granulopoiesis beyond stress-induced changes. The total proportion of monocytes remained unchanged following vortioxetine administration, but the fractions of Ly6C^low^ patrolling monocytes increased, while the fraction of Ly6C^high^ inflammatory monocytes remained stable. This suggests a redistribution within the monocyte population without altering the overall pool.

In contrast to both the vehicle and vortioxetine groups, treatment with the Vort:Na_2_GA (1:20) complex did not affect the total neutrophil fraction. However, a redistribution within the neutrophil population was observed: the proportion of functionally mature Ly6C^low^ neutrophils increased, accompanied by a trend toward a decrease in immature Ly6C^high^ forms (*p* = 0.0509). This may indicate accelerated neutrophil maturation without enhanced granulopoiesis. Concurrently, the complex reduced the proportion of inflammatory Ly6C^high^ monocytes, while Ly6C^low^ patrolling monocytes remained unchanged.

Thus, both drugs modulate the balance of monocyte subsets, albeit in opposite directions: vortioxetine increases the proportion of patrolling monocytes, whereas the complex reduces the fraction of inflammatory monocytes. These findings suggest that, unlike vortioxetine alone, the complex Vort:Na_2_GA (1:20) does not suppress granulopoiesis, promotes neutrophil maturation, and reduces inflammatory monocyte levels—potentially reflecting a more adaptive myeloid cell response to stress.

It is noteworthy that both vortioxetine and the complex mitigated the stress-induced alterations in T- and B-cell populations observed in the vehicle group (Figure S3).

### 3.7. The Effect of Vortioxetine and Its Complex on the Expression of Target Genes in Blood Cells of Intact Mice

**Figure 9:**
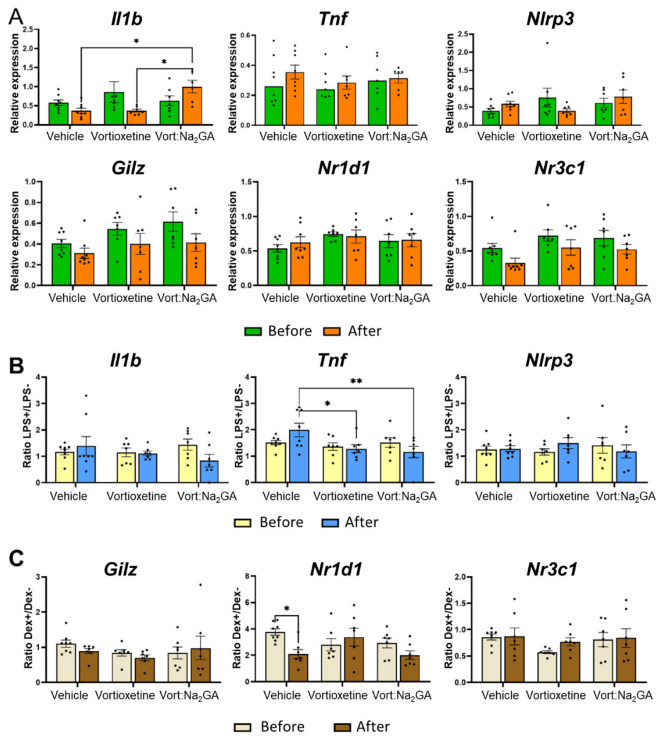
Impact of vortioxetine and its complex Vort:Na_2_GA (1:20) on gene expression in blood cells before and after 7 days of drug treatment. (**A**) Basal expression levels of genes (unstimulated blood cells). (**B**) Changes in pro-inflammatory gene expression in response to LPS treatment (LPS^+^/LPS^−^ ratio). (**C**) Changes in glucocorticoid-sensitive gene expression in response to dexamethasone treatment (Dex^+^/Dex^−^ ratio). Vehicle—group of mice receiving vehicle (N = 8); Vortioxetine—group of mice receiving vortioxetine (20 mg/kg) (N = 7); Vort:Na_2_GA—group of mice receiving the Vort:Na_2_GA (1:20) complex (20 mg/kg based on vortioxetine content) (N = 7). Blood samples were collected from each mouse before the start of treatment and 24 h after the final drug administration. Blood samples from each animal were treated ex vivo for 2 h with LPS (10 pg/µL), dexamethasone (5 ng/µL), or left untreated. * *p* < 0.05, ** *p* < 0.01; two-way repeated ANOVA with Sidak’s multiple comparisons test. Data are presented as mean ± SEM. Dots represent values from individual animals.

Pairwise post hoc comparisons revealed no differences between groups in the basal expression levels of the genes examined after 7 days of treatment, which is consistent with in vitro data obtained from SIM-A9 cells (Figure 9A). However, two-way repeated measures ANOVA revealed a significant main effect of time for the *Nr3c1* [F(1,18) = 11.5, *p* < 0.01] and *Gilz* [F(1,18) = 6.5, *p* < 0.05] genes: their expression decreased in all groups after treatment compared to baseline, likely due to the stressful procedures involved (oral gavage, blood collection).

Ex vivo analysis of blood cell responsiveness to LPS showed that the ratio of *Tnf* expression in stimulated versus unstimulated cells (LPS^+^/LPS^−^ ratio) was significantly reduced after administration of both vortioxetine and the complex compared to the control group (*p* < 0.05) (Figure 9B). This indicates modulation of the pro-inflammatory response by the tested compounds, although the effect was limited to the *Tnf* gene and did not extend to other pro-inflammatory genes examined.

Incubation of blood cells with dexamethasone for 2 h allowed us to assess glucocorticoid sensitivity after treatment. Administration of vortioxetine or the complex did not affect the sensitivity of *Nr3c1* expression to dexamethasone (Figure 9C). However, for the *Gilz* gene, a significant reduction in the inducible response was observed in the control group after 7 days of vehicle administration (*p* < 0.05), indicating the development of functional glucocorticoid resistance, likely related to the stress of treatment and blood collection. In contrast, in groups receiving vortioxetine or the complex, the response to dexamethasone remained unchanged, indicating that the compounds prevented the development of resistance. This effect is consistent with the findings described above for the SIM-A9 cell line, where the same compounds also contributed to overcoming glucocorticoid resistance.

Thus, the effect of the drugs on the LPS-induced response was limited and affected only the *Tnf* gene. At the same time, both compounds modulated dexamethasone sensitivity, preventing the development of functional resistance observed in the control group.

## 4. Discussion

### 4.1. Physicochemical Characterization of the Vort:Na_2_GA Complexes

Comprehensive physicochemical characterization confirms the successful formation of stable complexes between vortioxetine and the sodium salt of glycyrrhizic acid (Na_2_GA), and provides detailed insights into their structural properties. These findings are consistent with the studies demonstrating that mechanochemical technology is an effective approach for producing solid dispersions of drugs with polysaccharides and glycyrrhizic acid, resulting in loss of crystallinity and the formation of intermolecular complexes [31,36]. A significant increase in vortioxetine solubility was observed across all studied ratios, indicating the high efficacy of Na_2_GA as a complexing agent. Similar results have previously been reported for other lipophilic drugs, where complexation with glycyrrhizic acid led to a manifold increase in solubility and bioavailability [22,37]. Furthermore, incorporation of vortioxetine into Na_2_GA complexes enhanced its stability under accelerated storage conditions, likely by preventing oxidation of the molecule.

X-ray diffraction analysis and SEM revealed partial amorphization and particle size reduction (from 100 µm to 10–50 µm), facilitating molecular dispersion and distribution of vortioxetine within the excess solid carrier. These observations are consistent with the documented effects of mechanochemical treatment, which induce amorphization of crystalline structures and formation of solid dispersions [38,39,40].

^1^H NMR relaxation studies demonstrated a 50-fold reduction in T_2_ relaxation times of aromatic protons in the complexes (from 575 ms to ~10 ms), indicating incorporation into Na_2_GA micelles with biexponential decay kinetics. Comparison with the methanolic solution confirmed vortioxetine self-association in aqueous medium. These results are in good agreement with other studies employing NMR relaxation and diffusion measurements, which showed that glycyrrhizic acid forms self-associates in aqueous solutions and incorporates lipophilic drug molecules into its supramolecular structure [37,41]. Gel chromatography revealed an increase in the molecular weight of associates in the complexes, further supporting the micellar solubilization model.

Thus, mechanochemical treatment of vortioxetine with Na_2_GA enables the production of stable complexes with improved solubility, altered particle morphology, and complete incorporation of vortioxetine into the supramolecular structure of the carrier, which may significantly affect its pharmacokinetic properties and bioavailability.

The present study demonstrated that the developed molecular complex of vortioxetine with glycyrrhizic acid improves the pharmacological profile of the antidepressant. Incorporation of vortioxetine into the complex significantly reduced drug cytotoxicity. In the therapeutic concentration range (up to 7.5 μM), the Vort:Na_2_GA (1:20) complex exhibited no toxicity towards SIM-A9 microglial cells, whereas pure vortioxetine and complexes with lower GA content (1:5, 1:10) induced cytotoxicity. Even at toxic concentrations of vortioxetine (15 μM), the Vort:Na_2_GA (1:20) complex maintained cell viability at approximately 40% compared to ~10% in other groups. This effect correlates with data from other studies where glycyrrhizic acid demonstrated cytoprotective and membrane-stabilizing properties [23,24,41]. Furthermore, in a model of glucocorticoid overload (dexamethasone pretreatment) mimicking one of the pathophysiological mechanisms of depression [42,43], only the Vort:Na_2_GA (1:20) complex did not reduce cell viability, in contrast to pure vortioxetine. This indicates the potential of GA to mitigate negative cellular effects associated with both the drug itself and HPA axis hyperactivation.

In addition, studies on the Caco-2 intestinal epithelial model showed that the Vort:Na_2_GA (1:20) complex significantly increased the transport rate of vortioxetine across the cell monolayer compared to the free drug. This is consistent with the known ability of GA and its derivatives to act as permeability enhancers, potentially by inhibiting P-glycoprotein and/or affecting membrane integrity [44].

### 4.2. Anti-Inflammatory Effects of Vortioxetine and the Complex In Vitro and Ex Vivo

The primary therapeutic action of vortioxetine is mediated through the modulation of serotonergic neurons in the CNS. However, immune cells also express several subtypes of serotonin receptors, enabling direct crosstalk between the serotonergic system and inflammatory processes and making them targets for antidepressants [45,46,47]. Microglia express a wide range of serotonin receptors, including 5-HT_1_A, and these receptors are involved in regulating microglial activation and neuroinflammation [48]. Specifically, the presence of 5-HT_1_A receptors plays an important role in microglial cell survival upon inflammatory challenge [49].

The effects of vortioxetine on serotonergic receptors, specifically in microglia, have not been previously reported. Our results in SIM-A9 microglial cells show that 48 h treatment with vortioxetine alone weakly increases *Htr1a* gene expression, whereas the addition of GA, particularly at a 1:20 ratio, leads to a significant upregulation. Moreover, under conditions of glucocorticoid challenge (dexamethasone pretreatment), this effect was markedly potentiated, indicating that glucocorticoid stimulation sensitizes microglia to the action of the tested compounds, and that the Vort:Na_2_GA (1:20) complex may have an advantage over pure vortioxetine in modulating the serotonergic system at the microglial level. This may be particularly important in depression with HPA axis hyperactivity, where elevated glucocorticoids are common [42,43,50]. The complex’s ability to upregulate *Htr1a* specifically under glucocorticoid challenge could enhance serotonergic transmission and therapeutic efficacy in stress-related depressive disorders.

Our results revealed an anti-inflammatory profile for both vortioxetine and the Vort:Na_2_GA (1:20) complex in SIM-A9 microglial cells (in vitro) and in peripheral blood cells (ex vivo). Although the treatment regimens differed (48 h in vitro vs. 7 days of oral administration in mice), common features as well as tissue-specific aspects of the anti-inflammatory action were identified. A common finding was the lack of effect on basal expression of pro-inflammatory cytokine genes, indicating that the compounds do not cause non-specific immune activation in the absence of an inflammatory stimulus. This is in good agreement with literature data showing that antidepressants predominantly modulate the immune response under pathological conditions without disrupting basal homeostasis [51,52,53]. It has also been previously shown that vortioxetine does not alter basal expression levels of pro-inflammatory cytokines, both in vivo in various brain structures of intact animals following chronic administration [15,54,55] and in vitro in human monocyte cultures [14]. The anti-inflammatory properties of vortioxetine become evident under additional challenges that elevate cytokine levels, such as chronic stress [16,56] or LPS administration [14]. Importantly, the anti-inflammatory response does not affect the entire spectrum of cytokine expression but rather targets only select cytokines.

Consistent with the studies mentioned above, in our two experimental settings, the anti-inflammatory effect of vortioxetine and its complex was observed upon LPS-induced inflammation in microglia. In vitro, vortioxetine and the Vort:Na_2_GA (1:20) complex suppressed LPS-induced expression of *Nlrp3*, *Il1b*, and *Il6* genes, but did not affect *Tnf* gene expression. In contrast, in peripheral blood cells ex vivo, both compounds significantly reduced LPS-induced *Tnf* gene expression without altering other pro-inflammatory genes. This discrepancy may reflect differences in the kinetics and mechanisms of the inflammatory response in different cell types, as well as differences in the duration of compound and LPS exposure. Microglia and peripheral immune cells are known to differ in their signaling pathways and transcriptional regulation of pro-inflammatory genes, including the relative contributions of NF-κB-dependent and inflammasome-related mechanisms, which may determine the differential sensitivity of individual genes to pharmacological modulation [57,58]. Furthermore, the different time parameters likely contribute: with 24 h LPS exposure in vitro, compounds may preferentially affect early steps of inflammasome assembly and activation (*Nlrp3*/*Il1b*), whereas after 7 days of in vivo administration followed by 2 h ex vivo stimulation, regulation of the rapid TNF response predominates. Notably, the Vort:Na_2_GA (1:20) complex exhibited activity comparable to or greater than that of pure vortioxetine in both settings, confirming that complexation with GA preserves and in some cases enhances the pharmacological properties of the parent compound.

One possible molecular mechanism explaining the enhanced anti-inflammatory effect of the complex is the upregulation of *Nr1d1*, which encodes the transcription factor REV-ERBα. REV-ERBα is involved in restricting inflammatory responses in microglia by inhibiting NF-κB signaling [59] and directly suppressing *Il1b* activation by binding to its promoter [60]. The more pronounced anti-inflammatory effect of the complex may therefore be related to its ability to increase *Nr1d1* expression in microglia. In contrast, in peripheral blood cells in vivo, where REV-ERBα also participates in immune regulation [61], its expression is under the control of systemic factors (e.g., circadian rhythms or hormonal status), which may override the effect of our compound. Thus, the cell-specific action of the Vort:Na_2_GA complex on *Nr1d1* underscores its potential value specifically in the context of neuroinflammatory processes in the CNS.

In our experiment, although PCR analysis did not detect strong modulation of LPS-induced pro-inflammatory gene expression in whole blood after 7 days of drug treatment, flow cytometry revealed a redistribution of blood cell populations. The Vort:Na_2_GA (1:20) complex promoted an increase in the functionally mature Ly6C^low^ neutrophil subset and, most importantly, a reduction in the proportion of inflammatory Ly6C^high^ monocytes. These cells are key effectors of systemic inflammation, and their accumulation is directly associated with the development of depression-like behavior in preclinical models [62,63]. The observed changes in cell composition point to a longer-term mechanism of action of the complex. The complex likely modulates myelopoiesis, promoting the formation of a cell pool with a less pro-inflammatory phenotype, which is further supported by the preserved glucocorticoid sensitivity observed in gene expression analysis. These results indicate that the complex contributes to the establishment of a more sustained anti-inflammatory phenotype in vivo.

### 4.3. Modulation of Glucocorticoid Sensitivity

Chronic stress and the resulting depression are often accompanied by elevated glucocorticoid levels [42,43,50]. Prolonged elevation of glucocorticoids can lead to dysregulation of the HPA axis and the development of glucocorticoid (GC) resistance. GC resistance is frequently observed in depressed patients and in animal models of depression [42,64,65,66], particularly in immune cells [67,68]. Therefore, an important property of antidepressant drugs is the restoration of normal HPA axis function and the ability to overcome GC resistance [64].

In the present study, we demonstrated the effects of vortioxetine and its complex on the GR and GR-sensitive genes. Unlike pure vortioxetine, the Vort:Na_2_GA (1:20) complex significantly increased *Nr3c1* (*GR*) gene expression both in untreated SIM-A9 microglial cells and under dexamethasone treatment. This is an important distinction, as decreased GR expression and function have been demonstrated in chronic stress [69,70,71] and are associated with the development of GC resistance and the maintenance of chronic inflammation in depression [42]. This effect may be mediated by the presence of GA in the complex, as GA has been shown to modulate glucocorticoid signaling [26,30]. In peripheral blood cells from the in vivo experiment, however, we did not observe a significant change in basal GR expression, although a weak increase was seen for both compounds. Nevertheless, because the experiment was conducted in intact mice, no definitive conclusion can yet be drawn regarding the action of the complex under conditions of GC resistance in vivo.

More pronounced upregulation of GR-sensitive genes (*Fkbp5* and *Gilz*) in SIM-A9 microglial cells by the Vort:Na_2_GA (1:20) complex compared to vortioxetine and control, under dexamethasone pretreatment, confirms the preservation of glucocorticoid sensitivity even under conditions of chronic glucocorticoid stimulation that induced resistance in the control group. A similar situation was observed in blood cells from intact animals: both vortioxetine and the complex maintained the *Gilz* response to dexamethasone, whereas in control animals, the mild stress associated with the 7-day oral administration and blood collection significantly reduced this response. These data indicate that the tested compounds, particularly the developed complex, support glucocorticoid signaling under conditions that normally lead to the development of resistance.

Literature data support the ability of vortioxetine to modulate HPA axis function, which may contribute to its therapeutic action in depressive disorders associated with impaired glucocorticoid signaling. Brivio and colleagues [72] showed that chronic vortioxetine administration (14 days) to intact rats does not alter basal expression of GR or GR-sensitive genes but significantly enhances GR nuclear translocation in response to acute stress and increases expression of GR-sensitive genes. These findings are directly consistent with our results, demonstrating that vortioxetine does not affect basal transcription but preserves and even enhances glucocorticoid sensitivity under stress stimulation. Furthermore, in animal models of depression, vortioxetine normalizes elevated blood corticosterone levels [16], indicating restoration of HPA axis homeostasis. Notably, in depressed patients, chronic 8-week vortioxetine administration did not alter basal expression of GR or GR-sensitive genes [73], confirming the safety of the drug and the absence of undesirable chronic activation of glucocorticoid signaling. Collectively, these data suggest that vortioxetine, without disrupting basal HPA axis homeostasis, effectively modulates its reactivity under stress and may be a promising agent for correcting conditions associated with GC resistance.

When analyzing the effects of the complex, synergy between vortioxetine and GA can be proposed: vortioxetine may improve the functional state of GR (enhancing its nuclear translocation in response to a stimulus), whereas GA may reduce molecular disruptions in GR signaling caused by oxidative stress (e.g., by lowering ROS levels and inhibiting the p38 MAPK pathway) [24,26]. This explains why the complex shows superiority over pure vortioxetine in the SIM-A9 model, where chronic dexamethasone stimulation mimics a stress state leading to GC resistance. In contrast, in peripheral blood cells from intact animals, where the stress burden (gavage and blood collection) was milder and did not cause significant impairment, both compounds worked with comparable efficacy.

Our data showed that vortioxetine alone significantly increased the expression of *Abcb1a*, which encodes the efflux pump P-glycoprotein (P-gp), in SIM-A9 microglial cells. This could potentially reduce its intracellular concentration and efficacy [74,75]. The Vort:Na_2_GA (1:20) complex attenuated this induction, indicating that GA can modulate the undesirable effects of vortioxetine on transport systems.

## 5. Conclusions

In summary, the developed complex of vortioxetine with glycyrrhizic acid at a 1:20 weight ratio represents a promising formulation with an improved biological profile. It combines reduced cytotoxicity (particularly relevant in overdose situations and under glucocorticoid challenge) compared to pure vortioxetine, as well as faster permeability across epithelial cells in vitro. The complex exhibited enhanced stimulation of *5-HT_1_A* receptors, a key therapeutic target of the antidepressant, especially under conditions of elevated glucocorticoid load, relative to pure vortioxetine. Furthermore, the complex showed a greater ability than vortioxetine alone to upregulate GR expression and the expression of GR-sensitive genes (Fkbp5 and Gilz) under conditions of dexamethasone-induced GR overstimulation and developing GR resistance, suggesting its potential to overcome glucocorticoid resistance. However, this hypothesis requires further validation in vivo.

The anti-inflammatory effects of the complex on microglial cell cultures were more pronounced compared to those of pure vortioxetine, possibly due to the upregulation of Nr1d1, a transcription factor involved in immune response regulation. In contrast, on peripheral blood cells, the anti-inflammatory properties of the complex and vortioxetine were largely comparable, with the exception of a significant reduction in the proportion of inflammatory Ly6Chigh monocytes induced by the complex.

Thus, the developed Vort:Na_2_GA (1:20) complex exerts stronger effects on several molecular targets of vortioxetine, which can be attributed to the synergistic action of the components. Although we did not separately evaluate the effects of glycyrrhizic acid alone, the complex differs from vortioxetine only by the presence of GA; therefore, the observed differences can be explained either by GA itself or by the novel properties acquired through complex formation. A key aspect is the enhanced effects of the complex under conditions of heightened glucocorticoid activation, as well as its pronounced anti-inflammatory action on microglial cells. Further studies in animal models of depression will be necessary to assess whether the complex potentiates the antidepressant properties of vortioxetine, particularly in cases where depression is combined with an inflammatory component and HPA axis dysfunction.

Overall, the Vort:Na_2_GA (1:20) complex shows an improved biological profile relative to vortioxetine alone, warranting further in vivo investigation in depression models, especially those characterized by inflammation and HPA axis dysregulation.

## Figures and Tables

**Table 1:** Solubility of vortioxetine.

Sample	Time of Treatment	Solubility of Vortioxetine in Water, g/L	Stability of Vortioxetine, %
vortioxetine		1.6	-
Vort:Na_2_GA 1:5	2 h	>10	100.1 ± 1.1
Vort:Na_2_GA 1:10	2 h	>10	100.0 ± 2.3
Vort:Na_2_GA 1:20	2 h	>10	100.2 ± 1.5
Vort:Na_2_GA 1:40	2 h	>10	99.9 ± 2.0

Notes: The experiments were performed in triplicate, and the results were presented as mean ± standard deviation (SD).

**Table 2:** Accelerated storage data.

Sample	Stability of Vortioxetine, %			
	Original	After 30 Day	After 60 Day	After 90 Day
Vortioxetine	100.0 ± 1.3	99.1 ± 1.5	99.0 ± 2.5	89.1 ± 3.0
Vort:Na_2_GA 1:5	100.1 ± 1.1	99.8 ± 2.3	99.2 ± 1.5	96.9 ± 2.5
Vort:Na_2_GA 1:10	100.0 ± 2.3	99.5 ± 1.8	99.3 ± 1.1	97.7 ± 1.2
Vort:Na_2_GA 1:20	100.2 ± 1.5	99.3 ± 1.2	98.9 ± 2.3	97.8 ± 1.9
Vort:Na_2_GA 1:40	99.9 ± 2.0	99.7 ± 1.7	99.1 ± 1.3	98.2 ± 1.8

Notes: The experiments were performed in triplicate, and the results were presented as mean ± standard deviation (SD). Statistical significance was calculated using two-way ANOVA followed by Tukey’s HSD post hoc test.

## Data Availability

The original contributions presented in this study are included in the article/Supplementary Materials. Further inquiries can be directed to the corresponding authors.

## Supplementary Materials

The following supporting information can be downloaded at: https://www.mdpi.com/article/10.3390/biomedicines14071540/s1, Figure S1: Gating strategy for lymphoid cells; Figure S2: Gating strategy for myeloid cells; Figure S3: Analysis of lymphoid blood cell subpopulations before and after 7 days of drug treatment; Table S1: Primers and probes sequences.

## Abbreviations

GA	Glycyrrhizic Acid
GC resistance	glucocorticoid resistance
GPC	Gel Permeation Chromatography
GR	Glucocorticoid Receptor
HPA axis	Hypothalamic–Pituitary–Adrenal axis
HPLC	High-Performance Liquid Chromatography
LPS	Lipopolysaccharide
MWD	Molecular Weight Distribution
NMR	Nuclear Magnetic Resonance spectroscopy
SEM	Scanning Electron Microscopy
Vort:Na_2_GA	Complex Vortioxetine with Disodium Salt of Glycyrrhizic Acid
XRD	Powder X-ray Diffraction
