# Supplementary material for: A Vortioxetine–Glycyrrhizic Acid Supramolecular Complex: Synthesis and Cellular Effects on Microglial and Blood Cells Under Inflammatory and Glucocorticoid Challenge
Source: Biomedicines. 2026 Jul 9;14(7):1540. doi: 10.3390/biomedicines14071540 (PMC13406049; doi:10.3390/biomedicines14071540)
Supplement: Supplementary file 1 [file biomedicines-14-01540-s001.zip › Figure S1-S3.pdf]

Figure S1. Gating strategy for lymphoid cells.

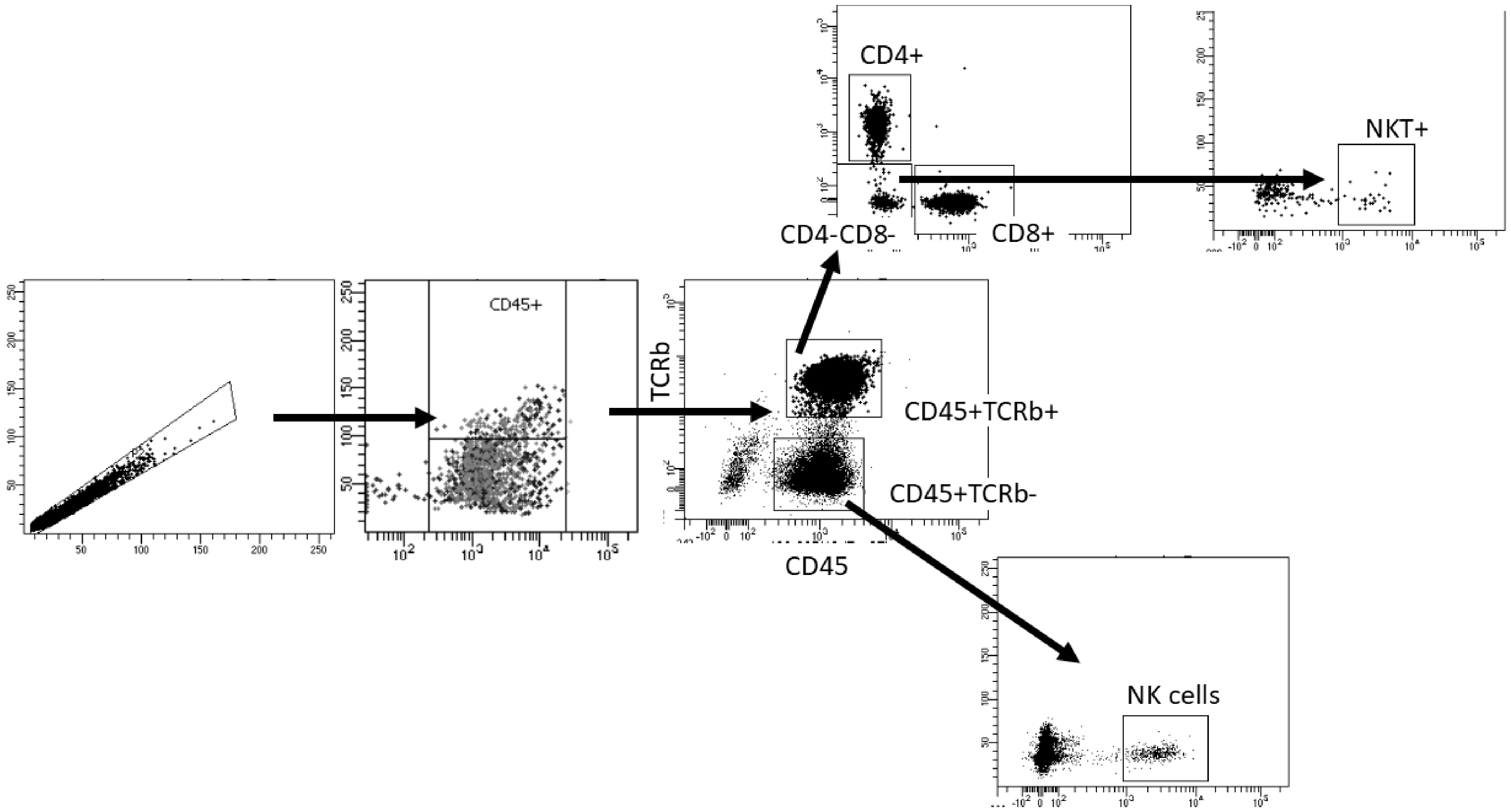

Figure S2. Gating strategy for myeloid cells.

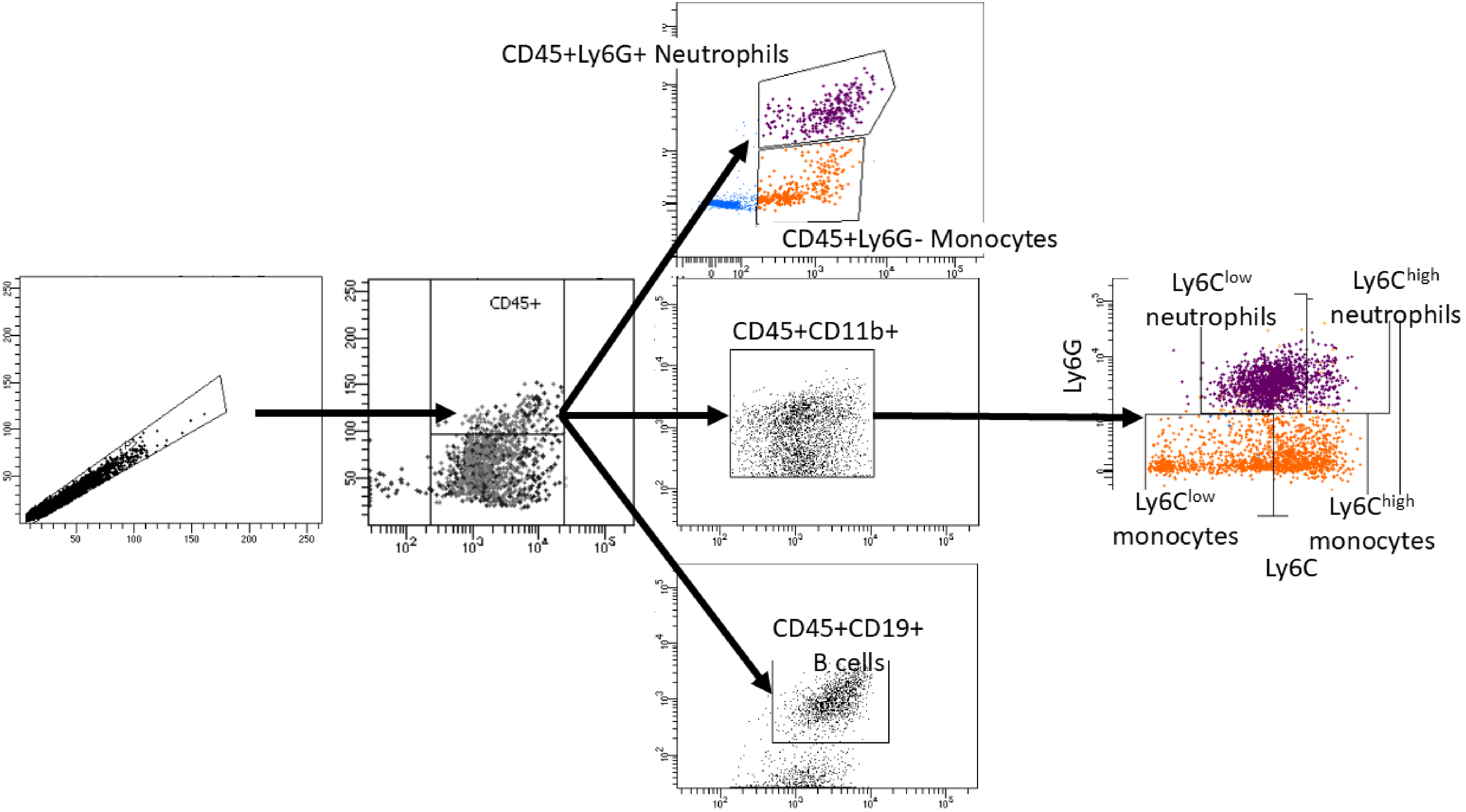

Figure S3. Analysis of lymphoid blood cell subpopulations before and after 7 days of drug treatment.

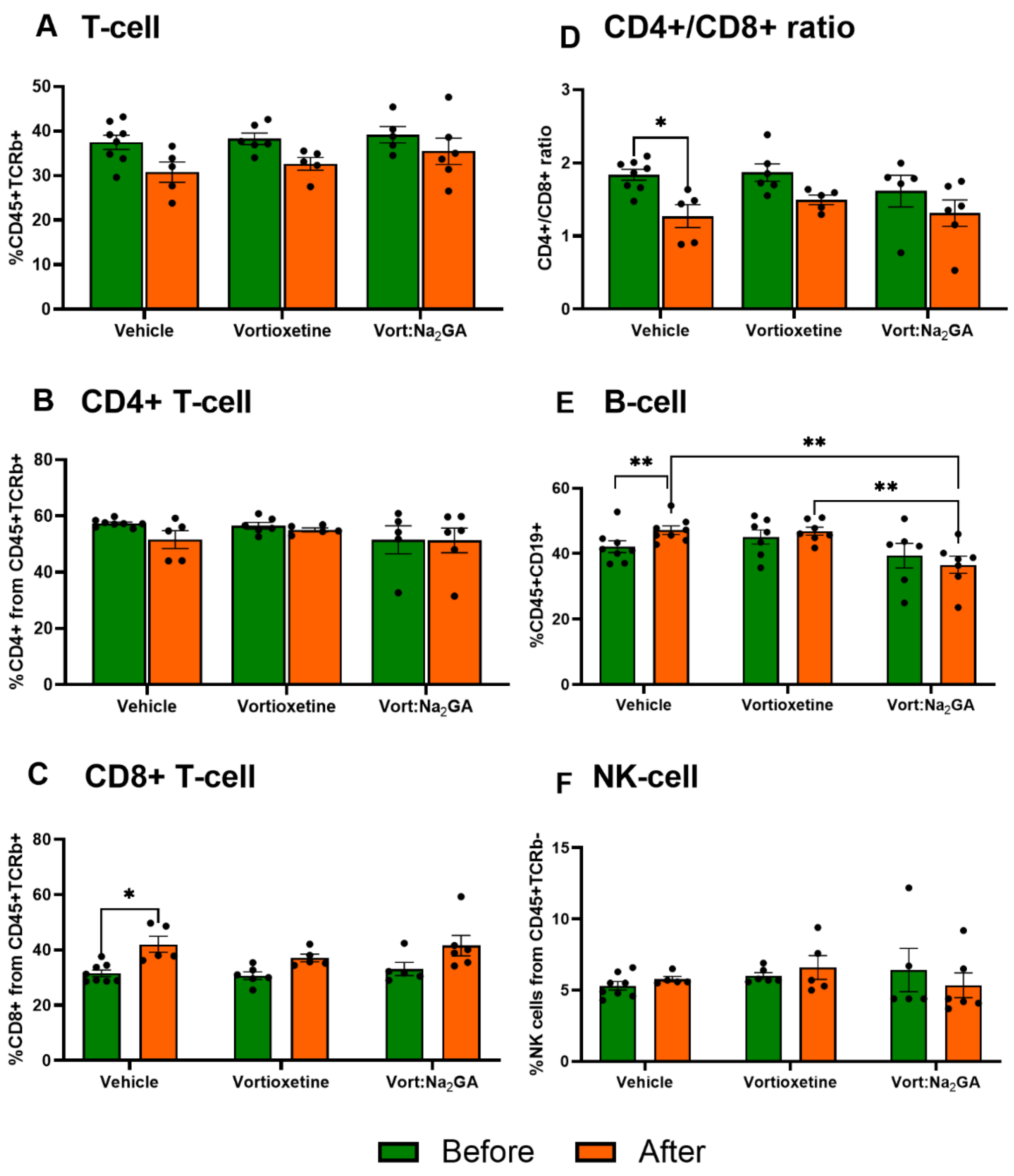

(A) Percentage of total blood T-lymphocytes (% CD45+TCRb+). (B) Percentage of CD4+ T-lymphocytes (from CD45+TCRb+). (C) Percentage of CD8+ T-lymphocytes (from CD45+TCRb+). (D) CD4+/CD8+ ratio. (E) Percentage CD19+ B-lymphocytes (from CD45+CD11b-). (F) Percentage of CD161+ NK cells (from CD45+TCRb-).

Vehicle - group of mice receiving vehicle (N = 8); Vortioxetine - group of mice receiving vortioxetine (20 mg/kg) (N = 7); Vort:Na_2GA - group of mice receiving the Vort:Na_2GA (1:20) complex (20 mg/kg based on vortioxetine content) (N = 7). Blood samples were collected from each mouse before the start of treatment and 24 hours after the final drug administration. * $p < 0.05$, ** $p < 0.01$; two-way repeated ANOVA with Šídák's multiple comparisons test.
