# Supplementary material for: A Vortioxetine–Glycyrrhizic Acid Supramolecular Complex: Synthesis and Cellular Effects on Microglial and Blood Cells Under Inflammatory and Glucocorticoid Challenge
Source: Biomedicines. 2026 Jul 9;14(7):1540. doi: 10.3390/biomedicines14071540 (PMC13406049; doi:10.3390/biomedicines14071540)
Supplement: Supplementary file 1 [file biomedicines-14-01540-s001.zip › Table S1.pdf]

Table S1. Primers and probes sequences.

Gene name	Gene Symbol	type	sequence 5' -> 3'	product size, bp
Interleukin 1 beta	*Il1b*	forward	CCTGTTCTTTGAAGTTGACGG	123
		reverse	CTGAAGCTCTTGTTGATGTGC	
		probe	ROX-CTGCTTCCAAACCTTTGACCTGG-BHQ2	
Interleukin 6	*Il6*	forward	CAGACCTGTCTATACCACTTCAC	236
		reverse	GGTACTCCAGAAGACCAGAGG	
		probe	ROX-CTGGGAAATCGTGGAAATGAG-BHQ2	
Tumor necrosis factor	*Tnf*	forward	CATCAGTTCTATGGCCCAGACCCT	101
		reverse	GCTCCTCCACTTGGTGGTTTGCTA	
		probe	ROX-CGAGTGACAAGCCTGTAGC-BHQ2	
NLR family, pyrin domain containing 3	*Nlrp3*	forward	CAGTGACAATACTCTGGGAG	116
		reverse	AGCATTGATGGGACAGTC	
		probe	ROX-TCTCTGAATGTTACAGCCTGGGT-BHQ2	
Nuclear receptor subfamily 1, group D, member 1	*Nr1d1*	forward	GCTGGTGAAGACATGACG	170
		reverse	GGGAAGTATGTGGGACAAC	
		probe	CCCTCTACAGTGACAGCTCCAAT	
Nuclear receptor subfamily 3, group C, member 1	*Nr3c1*	forward	ATGTATGACCAATGTAAACACA	132
		reverse	GCTCTTCAGACCTTCCTTAG	
		probe	ROX-TGCAGGTATCCTATGAAGAG-BHQ_2	
FK506 binding protein 5	*Fkbp5*	forward	AGAATCAAACGGAAAGGCGAG	103
		reverse	CTCGGCAATCAAATGTCCTTC	
		probe	ROX-CCAAACGAAGGAGCAACGG-BHQ_2	
TSC22 domain family, member 3 (Gilz)	*Tsc22d3*	forward	GTGGCCCTAGACAACAAGATT	122
		reverse	GAGTTCTTCTCAAGCAGCTCA	
		probe	TACGCTGTGAGAGAGGAGGTGGAG	
5-hydroxytryptamine (serotonin) receptor 1A	*Htr1a*	forward	GACTGCCACCCTCTGCCCTATATC	199
		reverse	TCAGCAAGGCAAACAATTCCAG	
		probe	ROX-ACGGCTGGTCTGGGAAAGTTGGAA-BHQ2	
ATP-binding cassette, sub-family B member 1A	*Abcb1a*	forward	CGATAAAAGAGCCATGTTTGC	138
		reverse	CTGATCTTGTGTATCTGTCTTCC	
		probe	ROX-TGCTGGTGTGCTCATAGTTGCCT-BHQ2	
Chaperonin containing TCP1 subunit 5	*Cct5*	forward	ACCAAACGGGCTGGA	238
		reverse	TCCTGGGATTTGGACAGT	
		probe	FAM-ACGATGGTGCCACCATTCTAAGCA-BHQ1	
Eukaryotic translation elongation factor 2	*Eef2*	forward	CCTGAAGCAGTTTGCGGAGA	93
		reverse	TCCTCTACTTTCTTGGCACGC	
		probe	Cy5-GGGCCAGCTGAGCGCAGCCG-BHQ2	
Phosphatidylinositol 3-kinase catalytic subunit type 3	*Pik3c3*	forward	GGATTGGCTGGACAGATT	108
		reverse	CTCCTTGTCATCGCACTT	
		probe	HEX-ACTTGATGGTTGAGTTTCGCTGTGT-BHQ1	
